# Yoganidra to Alleviate Anxiety: An Interventional Study

**DOI:** 10.7759/cureus.45083

**Published:** 2023-09-12

**Authors:** Prafull Kamble, Vandana S Daulatabad, Himaja Tandra, Anish Singhal, Madhusudhan U, Nitin A John

**Affiliations:** 1 Physiology, All India Institute of Medical Sciences, Bibinagar, IND; 2 Physiology, R.V.M. Institute of Medical Sciences and Research Center, Siddipet, IND

**Keywords:** anxiety scale, beck anxiety inventory, gad-7, physiological parameters, yoganidra

## Abstract

Introduction: Anxiety and stress are challenging conditions that result in perturbation of the body's homeostasis. It disturbs one's physical and mental state of equilibrium. There are many ways to overcome anxiety and stress, however, the best among many remedies is yoganidra, as it achieves optimum functioning of not only our body but also our mind. Hence, the present study was planned to evaluate the impact of yoganidra practice on the anxiety levels of undergraduate students.

Aims and objectives: This study aimed to evaluate physiological parameters like pulse rate, blood pressure, and respiratory rate, measure anxiety levels by the general anxiety disorder-7* *(GAD-7) inventory and Beck's anxiety questionnaire, conduct yoganidra sessions for all the students, and compare the effects of these training sessions on physiological parameters and anxiety scales.

Materials and methods: The present study was carried out in the department of physiology, R.V.M. Institute of Medical Sciences and Research Center, Siddipet, and conducted on 125 students from first to final Bachelor of Medicine, Bachelor of Surgery who participated voluntarily and actively after satisfying the inclusion and exclusion criteria. All the members were assessed for physiological cardio-respiratory parameters followed by the GAD-7 scale and Beck's anxiety questionnaire pre- and post-yoganidra sessions.

Results: There was a significant difference in blood pressure before and after the session, whereas the pulse rate and respiratory rate reduction after the session were highly significant. There was a highly significant reduction in GAD-7 anxiety score, from 12±3.41 to 5.80±2.56 (p<0.0001); while for Beck's score, there was a highly significant reduction in anxiety score of 20.83±0.73 after giving yoganidra training as opposed to the earlier score of 23.75±1.86 (p<0.0001).

Conclusion: Yoganidra provides ways to manage stress and anxiety and enhance mental wellness. It is supported by research evidence as a safe and effective method to reduce anxiety.

## Introduction

Anxiety is an experience categorized by fearful anticipation of an unpleasant event in the future. Stress is a challenging condition that results in perturbation of the body's homeostatic mechanism. Stress and anxiety are a state of mental or emotional brain conditions resulting from adverse demanding circumstances. Anxiety among medical students warrants greater attention due to its significant implications. The global prevalence rate of anxiety among medical students was 33.8% (95% CI: 29.2-38.7%) [1]. It disturbs one's physical and mental state of equilibrium causing other mental turbulences that might evolve into chronic conditions like hypertension, asthma, diabetes mellitus, and coronary heart disease [2,3]. Stress accelerates the nervous system, overburdens the adrenal glands, and lowers immunity [4,5]. Previous studies on medical students have shown that stress-induced disorders are higher in medical students than other age-controlled students [6].

Literature indicates that medical students undergo stress and anxiety due to exams, postings, other curriculum tasks, submissions, and high competitiveness. To overcome these effects of stress and anxiety, there is one among many remedies called yoganidra. Yoganidra is a psychic sleep or deep consciousness function awareness, where one is in deep relaxation, but consciousness is working at a deeper level. In psychology, the state achieved in yoganidra is termed the hypnogogic state, a state between sleep and wakefulness. The word "yoganidra" is derived from two Sanskrit words: "yoga" meaning union or integration and "nidra" meaning sleep. In this practice, the practitioner lies down in a comfortable, supine position while the instructor guides them through a systemic meditation. Yoganidra has six stages in it: internalization, sankalpa, rotation of consciousness of different parts of the body, breath awareness, opposite feeling and sensation, and externalization [7]. Yoganidra as envisaged in Indian scriptures is an anxiety-relieving tool for spiritual experience and is usually practiced for relaxation of body and mind for achieving good health. Yoganidra also helps spiritual seekers look for inner peace and acts as an alternative method to increase strength and endurance [8]. Yoga can achieve optimum functioning of not only the body but also the mind. It has only newly gained popularity worldwide [9]. When practiced regularly, it bestows physical health (as in cases of bronchitis, fibromyalgia, and menstrual disorders), mental health, and intellectual health [10-12]. It is found to be the most effective and useful practice for subjective well-being [13]. The benefits of yoganidra include stress reduction, improved sleep quality, enhanced focus and concentration, better emotional regulation, and an overall sense of well-being. It is particularly beneficial for individuals dealing with anxiety, insomnia, and other stress-related issues. Hence, the present study is intended to evaluate the impact of yoganidra training on anxiety levels in medical undergraduate students.

## Materials and methods

The present study was conducted in the department of physiology of R.V.M. Institute of Medical Sciences and Research Center, Siddipet, after obtaining ethical approval (2022/09), in which 125 volunteer students (convenience sample size) aged between 18 and 25 years were recruited for the interventional study after meeting the inclusion and exclusion criteria.

The inclusion criteria included students who have not practiced yoga and yoganidra sessions in the past six months and students aged between 18 and 25 years of both genders. The exclusion criteria included students with a history of previous or current organic diseases, hypertension, and diabetes mellitus, students who practiced yoganidra in the past three months, students with psychiatric disorders, and students who are addicted to smoking and alcohol, among others.

The relevance and purpose of the study were explained to all the students, and informed consent for participation in the study was obtained. They had their cardio-respiratory parameters like pulse rate, respiratory rate, and blood pressure checked, along with the generalized anxiety disorder-7 (GAD-7) [14] and Beck's anxiety questionnaire [15]. They were asked to answer openly and were explained about it confidentially. The students were informed about the program, made comfortable, and given orientation for the initial three to five days.

Yoga training was given for one hour (4-5 p.m.) five days a week for 12 weeks physically by a yoga teacher in a large yoga room. The session began with a yoga prayer, and mantras were chanted to connect with the inner divine and let go of negativity, and then two sets of Surya Namaskar with 12 steps as a warm-up session was practiced followed by meditative asana like vajrasana, padmasana, simhasana, and swastikasana. After these warm-up asanas, a yoganidra training session for 30 minutes was conducted. The yoganidra session was conducted in the following stages: The first stage is internalization, in which the initial relaxation of the body and mind was brought by inducing the awareness of stillness and comfort of the body by correcting posture and position and speed of breath and listening to the external sounds. They were made aware of the surroundings by instructing them to be in the state of witnessing the surrounding sounds and activity. In the second stage of sankalpa, the practitioners were instructed to take sankalpa or a resolution intention to enter into the practice of yoganidra: "I will not sleep" or "I will remain awake." During the third stage, consciousness or awareness was systematically switched throughout the different parts of the body. Subjects were instructed to remain aware, listen to the instructions, and very rapidly move their mind according to the instructions without making any physical movements. A definite sequence was followed to shift the awareness. In the fourth stage, the subjects were asked to become conscious of their natural breath without changing their breath flow. The subjects were aware of each inhalation and exhalation by mentally counting them. During the fifth stage, the physical or emotional sensations were recalled, intensified, and fully experienced. Pairs of contradictory feelings or sensations were practiced by asking subjects to imagine heat and cold, heaviness and lightness, pain and pleasure, love and hate, and so on. In the sixth stage of visualization, the subjects were asked to visualize some objects, stories, or situations in the chidakasha (“space of consciousness” or “inner space). At this time once again, the subjects were asked to mentally repeat sankalpa, which was taken earlier in stage two, with full dedication, faith, and optimism. In the seventh and final stage, slowly, the awareness was externalized by asking the subjects to become aware of the external sounds, objects, and persons. They were then asked to slowly move their body parts and stretch their body.

Initially, for a few days, we observed that most of the subjects fell asleep for 5-10 minutes. It was difficult for them to remain awake during yoganidra. A few days later, they got used to the practice of yoganidra. After the yoganidra training for 12 weeks, all baseline parameters along with GAD-7 and Beck's anxiety scales were obtained from all the participants, and the baseline versus postintervention scores were analyzed.

To assess the general anxiety disorder, a questionnaire of seven different questions was included to assess individual general anxiety levels. The total score was graded from 0 to 21. The score was categorized into low (<10), moderate (11-20), and severe (>21). Beck's anxiety inventory contained 22 types of common anxiety symptom questions. It measures the current level of anxiety. A total score of 0-21 indicates very low anxiety, 22-35 moderate anxiety, and >36 severe anxiety. For the statistical analysis, descriptive statistics like mean and standard deviation were calculated for all the parameters recorded, and a comparison of scores after giving an intervention of yoganidra training was analyzed using paired t-test with SPSS Statistics version 20 (IBM Corp. Released 2011. IBM SPSS Statistics for Windows, Version 20.0. Armonk, NY: IBM Corp.). A p-value of <0.0001 was considered highly significant.

## Results

**Figure 1:**
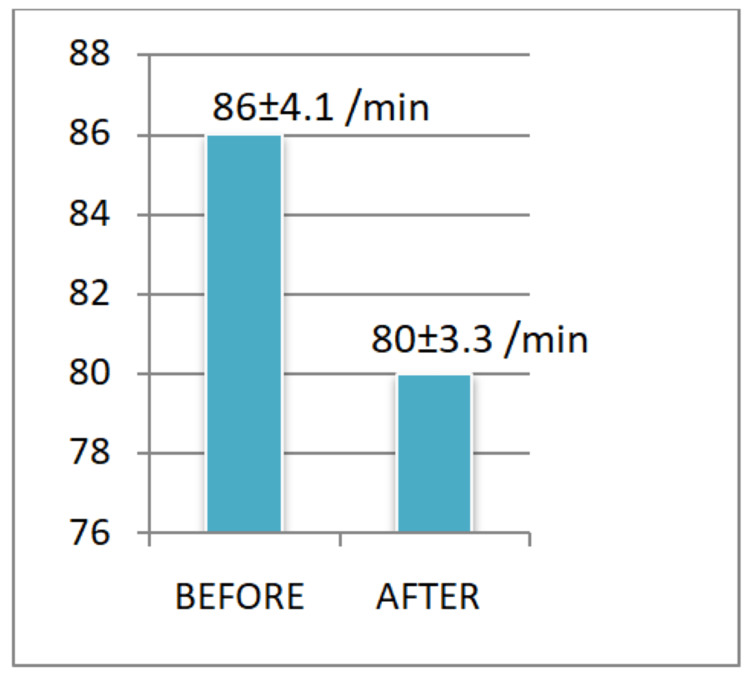
Pulse rate before and after the yoganidra sessions The pre-post difference in the pulse rate of the study participants is highly significant.

**Figure 2:**
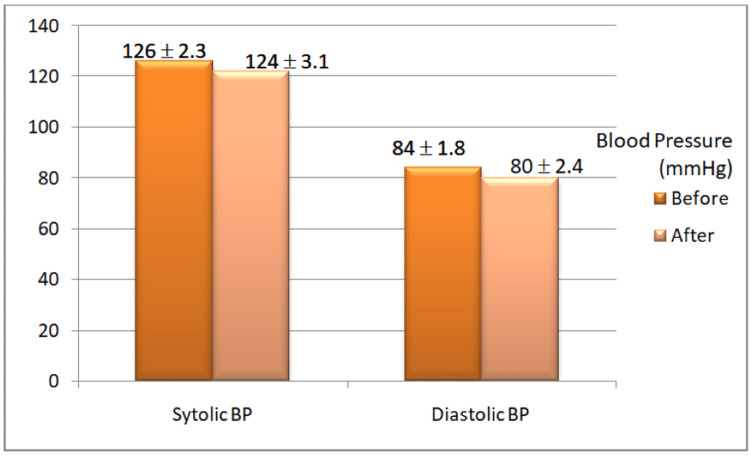
Blood pressure measurements before and after the yoganidra sessions Systolic and diastolic blood pressure readings after the yoganidra intervention decreased significantly.

**Figure 3:**
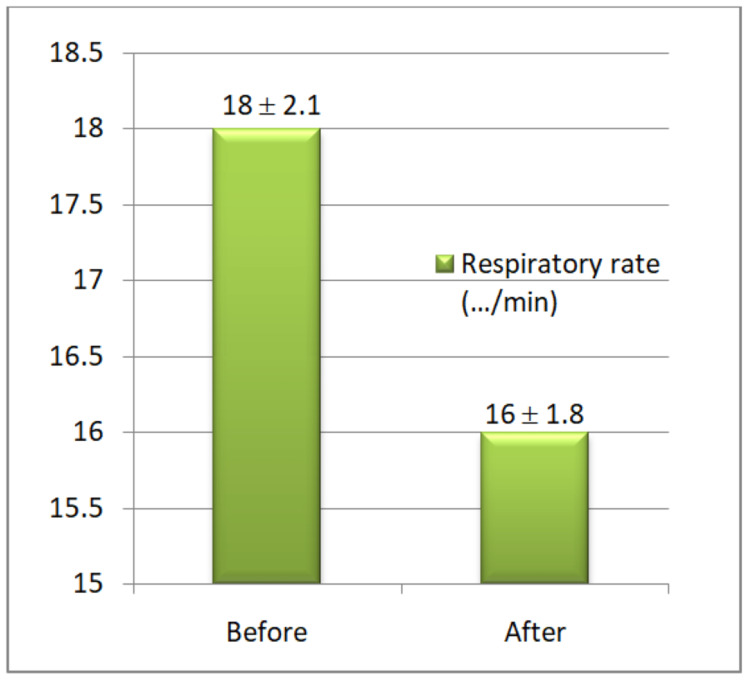
Respiratory rate changes before and after the yoganidra sessions Baseline and post-intervention readings of respiratory rate were highly significant.

**Figure 4:**
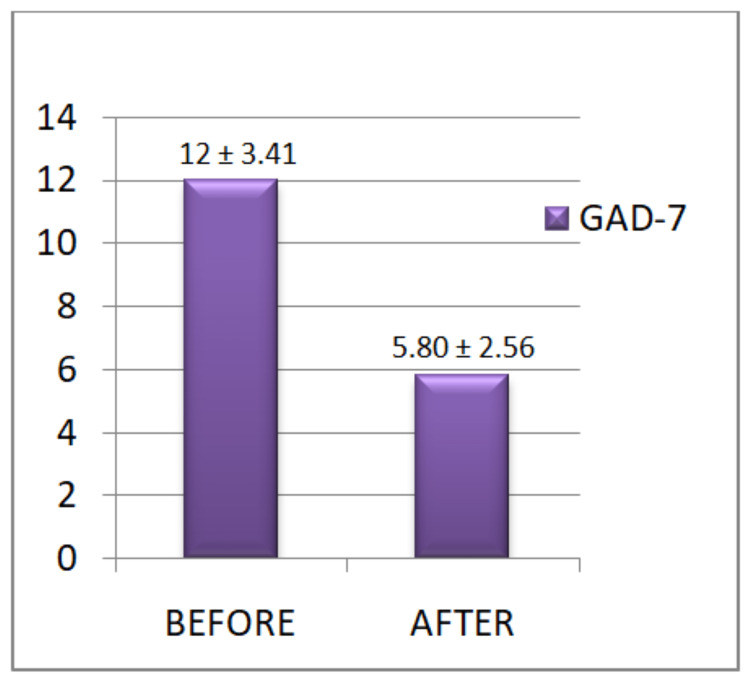
GAD-7 results before and after the yoganidra sessions Pre-intervention, the students were found to have moderate anxiety level scores, which significantly dropped down to low anxiety score levels after the yoganidra intervention.

**Figure 5:**
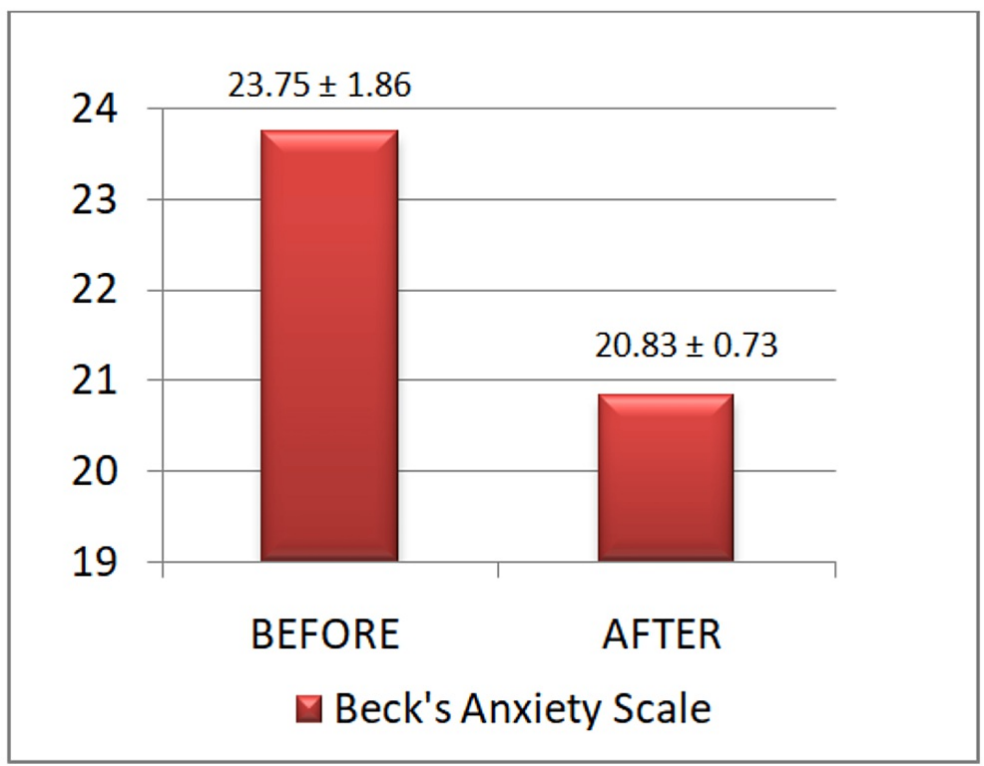
Beck's anxiety scale before and after the yoganidra sessions Students showed a significant reduction in anxiety levels from moderate to low.

**Figure 6:**
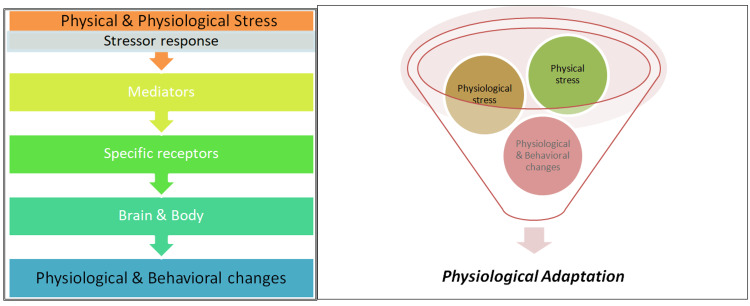
Flowchart of the physiology of stress Image credit: Dr. Prafull

Our results depict that medical undergraduate students were under moderate stress and anxiety at the beginning of the study. After the completion of intervention by yoganidra training sessions, the anxiety levels dropped from moderate to mild or low levels along with a decrease in baseline cardio-respiratory parameters like pulse rate, blood pressure, and respiratory rate (Table 1, Figures 1-6).

**Table 1:** Parameters before and after yoganidra training intervention The above results show that medical undergraduate students had moderate anxiety (GAD-7 12±3.41 and Beck's 23.75±1.86) at the start of the study. After intervention by yoganidra, their anxiety levels dropped from moderate to mild or low levels (GAD-7 5.8±2.5 and Beck's 20.83±0.73) along with a decrease in baseline cardio-respiratory parameters like pulse rate, blood pressure, and respiratory rate. There was a significant statistical difference in anxiety levels before and after yoganidra in the given parameters.

	Pulse rate (beats/min)	Blood pressure (mmHg)		Respiratory rate (rate /min)	GAD-7 scale score	Beck’s scale score
		Systolic	Diastolic			
Before (mean ± SD)	86 ± 4.1	126 ± 2.3	84 ± 1.8	18 ± 2.1	12 ± 3.41	23.75 ± 1.86
After (mean ± SD)	80 ± 3.3	124 ± 3.1	80 ±2.4	16 ± 1.8	5.8 ± 2.5	20.83 ± 0.73

## Discussion

The results of the study revealed that yoganidra reduces anxiety by activating the parasympathetic nervous system and inhibiting the sympathetic nervous system, thus promoting relaxation as reflected in the above results, i.e., a decrease in the cardio-respiratory parameters and anxiety scales. It also demonstrated that yoganidra benefits alleviate anxiety, improve concentration, and contribute to healthy living. Additionally, the practice encourages mindfulness and self-awareness, helping individuals manage their thoughts and emotions more effectively. In stressful conditions leading to highly anxious states, there will be a decrease in the activity of the parasympathetic system and a preponderance of the sympathetic activity. These all things happen due to the stress-induced imbalance in the body. As shown in Figure 6, physiological and physical stressors induce stress response to the hypothalamus and sympathetic axis from where mediators act on the body and brain as energy metabolism, metabolic change, immune reactivity, etc., and finally adaptation takes place. The increase in blood pressure, pulse rate, and respiratory rate is due to the increase in sympathetic functioning and the decrease in parasympathetic tone.

As per available literature, the physiological basis for how yoganidra relieves anxiety levels is due to the generation of alpha-wave frequencies in the cortex by the hypothalamus which becomes the underlying mechanism for calmness in people who practice yoga [16]. Generally, in the body, GABA levels are increased by the raised phenylalanine levels which lead to balanced mental activity and also an increase in plasma cortisol to combat stress, while low GABA levels are associated with higher anxiety [17]. the hypothalamic-pituitary-adrenal axis and sympathetic nervous systems are downregulated, with a tendency for the para-sympathetic nervous system to predominate. As a result, blood pressure, pulse rate, and respiratory rate are reduced. Yoga practices balance all systems of the body, lowering anxiety and improving overall mood resulting in increased mental clarity, emotional stability, and a greater sense of well-being [18,19]. Yoganidra and meditative asanas also help decrease arousal, reduce anxiety, improve concentration, and keep the person focused [20]. Our findings are in line with a study by Sharma et al. that found a significant reduction in stress, anxiety, and depression scores from baseline assessment when combined with conventional treatment using yoganidra [21]. According to Prafull et al.'s study, patients with the omicron variant reported symptoms like excessive fatigue and mild muscle aches. which also have a connection to stress and have an impact on hypothalamic function [22,23]. A study published in the Indian Journal of Physiology and Pharmacology found that yoganidra practice led to better sleep parameters and increased total sleep time in participants with sleep disturbances. A study published in the Journal of the International Society Psychological Regulation found that yoganidra was effective in reducing post-traumatic stress disorder (PTSD) symptoms and improving the quality of life in veterans with PTSD [24].

## Conclusions

Yoganidra provides solutions to reduce stress and anxiety and improve concentration. Yoganidra is supported by research evidence as a safe and effective method that everyone of all age groups can follow to reduce anxiety/ stress. Adapting and implementing the principle of yoganidra in day-to-day life may decrease the severity of anxiety and provide well-being to live. Yoganidra is also helpful in improving health and preventing degeneration, disease, and decay. It acts as a power nap to enhance the concentration levels of the individual. Thus, we could say yoganidra is a vaccine for the prevention of stress. It is a drug-less therapy for a germless disease like stress-induced anxiety.

When the students underwent yoganidra sessions for 12 weeks, their anxiety levels reduced from moderate to low levels. It is necessary for medical students to practice it daily to overcome stress and anxiety. In addition, the National Medical Council should include it in its daily schedule for the betterment of students.

Yoganidra should not be considered as a substitute for professional medical or psychological treatment. If you are interested in incorporating yoganidra into your routine, it is advisable to learn from a qualified instructor or use guided audio sessions. Furthermore, yoganidra's emphasis on imitation techniques offers stress and anxiety management techniques and is typically encountered early in the therapeutic process, igniting the desire for lasting change. Along with this comes the need for a readiness to be open to the idea that yoga practices could stand for paradigms and systems that, despite being different, are just as complex as the models used in modern science.
